# New Insights Into the Local Auxin Biosynthesis and Its Effects on the Rapid Growth of Moso Bamboo (*Phyllostachys edulis*)

**DOI:** 10.3389/fpls.2022.858686

**Published:** 2022-05-03

**Authors:** Yucong Bai, Miaomiao Cai, Changhong Mu, Wenlong Cheng, Huifang Zheng, Zhanchao Cheng, Juan Li, Shaohua Mu, Jian Gao

**Affiliations:** Key Laboratory of National Forestry and Grassland Administration/Beijing for Bamboo & Rattan Science and Technology, International Center for Bamboo and Rattan, Beijing, China

**Keywords:** auxin, YUCCA, local biosynthesis, rapid growth, culm sheaths, moso bamboo

## Abstract

Auxin plays a crucial regulatory role in higher plants, but systematic studies on the location of auxin local biosynthesis are rare in bamboo and other graminaceous plants. We studied moso bamboo (*Phyllostachys edulis*), which can grow up to 1 m/day and serves as a reference species for bamboo and other fast-growing species. We selected young tissues such as root tips, shoot tips, young culm sheaths, sheath blades, and internode divisions for local auxin biosynthesis site analysis. IAA immunofluorescence localization revealed that auxin was similarly distributed in different stages of 50-cm and 300-cm bamboo shoots. Shoot tips had the highest auxin content, and it may be the main site of auxin biosynthesis in the early stage of rapid growth. A total of 22 key genes in the YUCCA family for auxin biosynthesis were identified by genome-wide identification, and these had obvious tissue-specific and spatio-temporal expression patterns. *In situ* hybridization analysis revealed that the localization of *YUCCA* genes was highly consistent with the distribution of auxin. Six major auxin synthesis genes, *PheYUC3-1*, *PheYUC6-1*, Phe*YUC6-3*, *PheYUC9-1*, *PheYUC9-2*, and *PheYUC7-3*, were obtained that may have regulatory roles in auxin accumulation during moso bamboo growth. Culm sheaths were found to serve as the main local sites of auxin biosynthesis and the auxin required for internode elongation may be achieved mainly by auxin transport.

## Introduction

Bamboo has been reported to have more than 10,000 uses, and its value has reached $75 billion and continues to steadily increase (Gao et al., 2014, 2021). As the largest bamboo species in the world in terms of plantation area, moso bamboo (*Phyllostachys edulis*) is an important dual-purpose bamboo species for the production of bamboo shoots and timber in the subfamily Bamboo of the family Gramineae. Although it is a perennial plant, its shoot period is only 3 or 4 months, and as a perennial woody monocotyledonous plant of the family Gramineae, its growth and development are conservative and similar to those of other annual monocotyledonous herbaceous plants in the Gramineae. For example, it has the same internode development pattern as maize, and its auxin synthesis and regulation pathways are similar to those in rice (Zhang et al., 2014; Gao et al., 2021).

Bamboo is famous for its rapid growth. The fastest growth rate during the spring growth period can exceed 1 m per 24 h (Peng et al., 2013). Its growth pattern is similar to that of maize, which involves the control of internode length and the number of nodes by the regulation of internode meristem formation and rapid cell elongation. Multiple nodes elongate together to produce the high growth pattern. Its rapid growth has many regulatory processes such as sugar mechanical stress. Plant hormones, especially auxin, also play important roles (Li et al., 2018a; Wei et al., 2019; Gao et al., 2021; Wang et al., 2021). However, most studies on the regulation of auxin in moso bamboo have focused on the signal transduction process. Few studies have focused on auxin biosynthesis, and no studies have reported on synthesis sites (Wang et al., 2017; Li et al., 2018b). Auxin is probably synthesized in young tissues, such as stem tips, developing young leaves, and root tips (Ljung et al., 2001, 2005). In early developing spring shoots, there is no vegetative leaf structure, and its main plant structures include the bamboo culm, root, culm sheath (metamorphic sheath-like leaves attached to each node of bamboo shoots), and sheath blade (upper part of the bamboo culm sheaths). These sites are possible locations for auxin biosynthesis.

Auxin is critical in regulating the growth of higher plants. It does so by affecting cell elongation and cell differentiation and by synergistically regulating growth in combination with other plant hormones and sugars (Wong et al., 2009; Le et al., 2010; Perrot-Rechenmann, 2010). Auxin regulation acts mainly with active free IAA, while the fate of different IAA conjugates varies by species. IAA-alanine and IAA-leucine conjugates can be hydrolyzed to free IAA to remain active, while IAA-aspartate conjugates are usually not hydrolyzed (Westfall et al., 2010; Damodaran et al., 2017).

The auxin biosynthesis pathway is highly conserved in plants (Zhao, 2010). The main synthetic pathway of auxin has received the most study. The tryptophan-dependent indole pyruvate metabolic pathway is the main pathway of auxin synthesis, mainly through the conversion of tryptophan to indole pyruvate by tryptophan aminotransferase (TAA) and then through the rate-limiting irreversible reaction catalyzed by YUCCA-type flavin-containing monooxygenase (FMO): the oxidative decarboxylation of IPyA to IAA (Zhao et al., 2001; Zhao, 2018). In Arabidopsis, the tissue-specific expression of YUCCA proteins can be used as a marker for auxin distribution but not the widespread expression of TAA proteins. Thus, spatiotemporally similar co-expression of specific combinations of TAA and YUCCA members controlling local auxin biosynthesis is required to ensure partial organ development (Zhao, 2018; Blakeslee et al., 2019). The *YUCCA* gene family has been identified in 20 plant species by genome-wide evolutionary analysis, including the monocots rice and maize, the dicots Arabidopsis and cucumber, the early evolution plants *Physcomitrella patens* and *Marchantia polymorpha*, the conifer *Picea abies*, and the deciduous tree *Populus trichocarpa* (Cao et al., 2019).

The locations of auxin synthesis in bamboo plants have not been systematically determined. Young leaves and the stem tip are the main sites of auxin synthesis, but root tips can also synthesize auxin in *Arabidopsis thaliana* (Ljung et al., 2001, 2005; Wozniak et al., 2019). The regulation of auxin synthesis is complex, and local auxin biosynthesis and transport in multiple organs may be an important way to regulate growth (Friml et al., 2003; Ikeda et al., 2009; Kasahara, 2016). Therefore, we investigated the location of auxin biosynthesis in moso bamboo and resolved the spatio-temporal expression pattern of the *YUCCA* gene family in moso bamboo by bioinformatics and *in situ* hybridization, which provided a basis for understanding the local biosynthesis and regulation of auxin in moso bamboo and provided some reference value for the systematic study of auxin synthesis sites in graminaceous plants.

## Materials and Methods

### Plant Materials

All of the experimental materials were collected within the natural distribution area of moso bamboo in Guangde city, Anhui Province, China (30.89371 N, 119.41769 E). Spring shoots with good natural growth conditions were selected and collected at a height of 50 ± 2 cm and 300 ± 2 cm. After manual removal of the culm sheath, the split zone of young internodes 1/5 from the shoot tip (the 10th internode and the 18th internode up from the base), the sheath blade of the corresponding node, the young culm sheath of tip wrapped, the shoot tips, and the roots were selected (Supplementary Figure S1). Samples from three plants were taken as a mix, and six mixes were taken as biological replicates. Three replicates of three mixes were fixed in paraformaldehyde for sectioning, and the other was snap frozen in liquid nitrogen and stored at −80°C.

### Determination of Endogenous Auxin Content by High-Performance Liquid Chromatography-Mass Spectrometry

The sample was ground in liquid nitrogen, 80 ± 5 mg of the sample was taken in a 2-mL centrifuge tube, 50 μl of the internal standard solution, and 1 ml of aqueous acetonitrile (1% FA) were added, shaken, and then mixed for 2 min; the sample was extracted for 12 h at 4°C, protected from light, and centrifuged at 14,000 *g* for 10 min; then, 800 μl of the supernatant was taken, blow-dried with nitrogen, and re-dissolved with 200 μl of aqueous acetonitrile (1:1, v.v^−1^); and then, 14,000 *g* of the supernatant was centrifuged for 10 min, and the supernatant was extracted for chromatography-mass spectrometry analysis. The samples were separated using an Agilent 1290 Infinity LC ultra-high-performance liquid chromatography system and a Thermo Scientific TSQ Quantiva mass spectrometry system.

### IAA Immunofluorescence Localization Assay

Paraffin-fixed samples were vacuum pumped to allow complete liquid permeation and incubated overnight in the dark. The samples were then paraffin sectioned to a thickness of 8 μm (Hord et al., 2006; Wang et al., 2010). The sections were dewaxed and incubated again in paraformaldehyde and washed twice for 10 min, each with washing buffer (0.2 M phosphate buffer, 0.1% [vol.vol^−1^] Tween 20). The sections were then immersed in bovine serum protein (BSA) blocking solution for 1 h at room temperature, and the anti-IAA antibodies (BS-0902R) were incubated overnight at 4°C. After incubation, the samples were washed two or three times in 10 mM phosphate buffer and 0.1% (vol.vol^−1^) BSA for 10 min at a time and then washed with 10 mM phosphate-buffered saline (PBS) and 0.8% (wt.vol^−1^) BSA for 10 min. Anti-rabbit secondary antibody Fluor 488 was diluted in 1:500 blocking solution, incubated for 4 h at room temperature, protected from light, washed twice with 10 mM PBS, 0.88% (wt.vol^−1^) NaCl, 0.1% (vol.vol^−1^) Tween 20, and 0.8% (wt/vol^−1^) BSA for 15 min each time, and then washed for 1 min with 10 mM PBS and blocked with glycerol (Zheng et al., 2021). After Zeiss fluorescence microscopy imaging (Axio Imager D2), final editing was performed using Photoshop 2019. All of the experiments were performed in three biological replicates, and similar results were obtained.

### Identification and Screening of *YUCCA* Gene Family

Using the YUCCA sequence retrieved from the Arabidopsis^1^ rice^2^ databases as a reference, the genomic data downloaded from the moso bamboo Genome Database (BambooGDB)^3^ were blast analyzed. All sequences with *e* values ≤10^−11^ and scores ≥100 were used as new queries for the second round-robin search to avoid missing additional orthologs. The protein structural domains were analyzed using the Pfam program^4^ and SMART^5^ to ensure that the correct sequences were obtained. Multiple sequence analysis was performed using the ClustalW default program. A phylogenetic tree of the above protein sequences was constructed by using the MEGA6 program^6^ with the neighbor-joining (NJ) method, and 1,000 replicates were performed for the bootstrap analysis (Larkin et al., 2007; Tamura et al., 2013).

### RNA Extraction and qRT-PCR

Total RNA was extracted using the RNAprep pure Plant Kit (TIANGEN, DP432) and reverse transcription using the EvoM-MLV RT Kit with gDNA Clean for qPCR (Accurate Biology, AG11705). The quantitative real-time PCR (qPCR) was performed using the 2× All-in-one ™-qPCR Mix (Genecopoeia, Qp001-01). The specific primers were designed using Primer 3 Input software (version 4.1.0). The internal reference gene was *TIP41* (Fan et al., 2013); primers are shown in the Supplementary Table S4. Expression patterns were analyzed by transcriptomic data and real-time quantitative PCR to synthetically screen candidate tissue-specific genes for *in situ* hybridization analysis (Wang et al., 2017; Li et al., 2018a). Gene expression heat mapping was performed using TBtools software (Chen et al., 2020).

### *In situ* Hybridization

Related samples were fixed in 4% paraformaldehyde for 24 h, subjected to 10-μm paraffin sectioning, and affixed to lysine-containing slides for subsequent dehydration, baking, and dewaxing (Hord et al., 2006). *In situ* probe (antisense and sense) PCR amplification of the screened *YUCCA* gene was performed using T7 and SP6 RNA polymerase binding site primers. The hybridization signal was detected with anti-digoxin antibody coupled to NBT/BCIP solution.

## Results

### Multi-Organ Specific Distribution of Auxin in the Pre-rapid Growth Period

**Figure 1:**
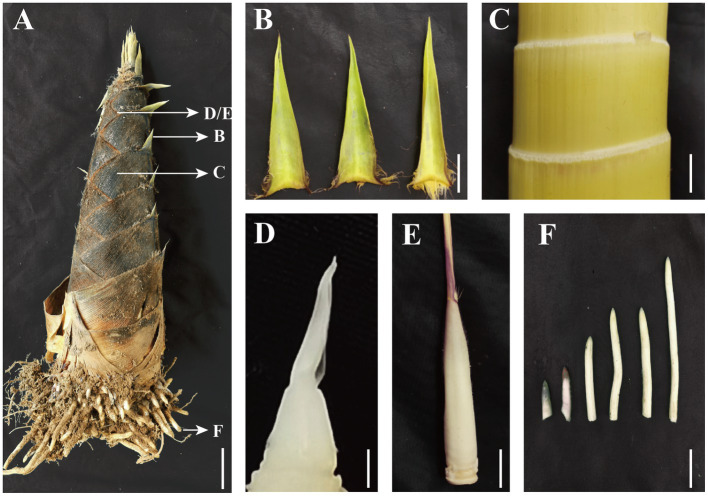
Structure of moso bamboo shoots with corresponding sampling tissues **(A)** 50-cm-height moso bamboo shoot, **(B)** sheath blade (the upper component of culm sheaths, the base is connected to the tip of sheath and is the metamorphic leaf of bamboo shoot), **(C)** bamboo shoot internode 1/5 from the tip, **(D)** young sheath wrapped shoot tip, **(E)** intact young sheath, **(F)** root tip (the tip of the bamboo root growing out of the base of the bamboo shoot). **(A)** Bar = 5 cm, **(B–F)** Bar = 1 cm.

Auxin is essential for rapid growth and for regulation of the underground shoot growth and freshly emerged growth stages of moso bamboo (Shou et al., 2020). According to the research of our group, for moso bamboo, 50-cm bamboo shoot is the state when it just emerges and starts to grow, while 300-cm bamboo shoot is the state when it enters the rapid growth stage (Li et al., 2018a). It is important to study the local biosynthetic accumulation of auxin in the early stage of rapid shoot growth to determine the regulatory role of the auxin. We selected all young parts of spring bamboo shoots of about 50 and 300 cm height (Figure 1A; Supplementary Figure S1), including the roots (Figure 1F), the split zone at internode 1/5 height from the shoot tip (Figure 1C), the sheath blade of the corresponding nodes (Figure 1B), the shoot tips (Figure 1D), and the culm sheath wrapping the shoot tip (Figure 1E).

**Figure 2:**
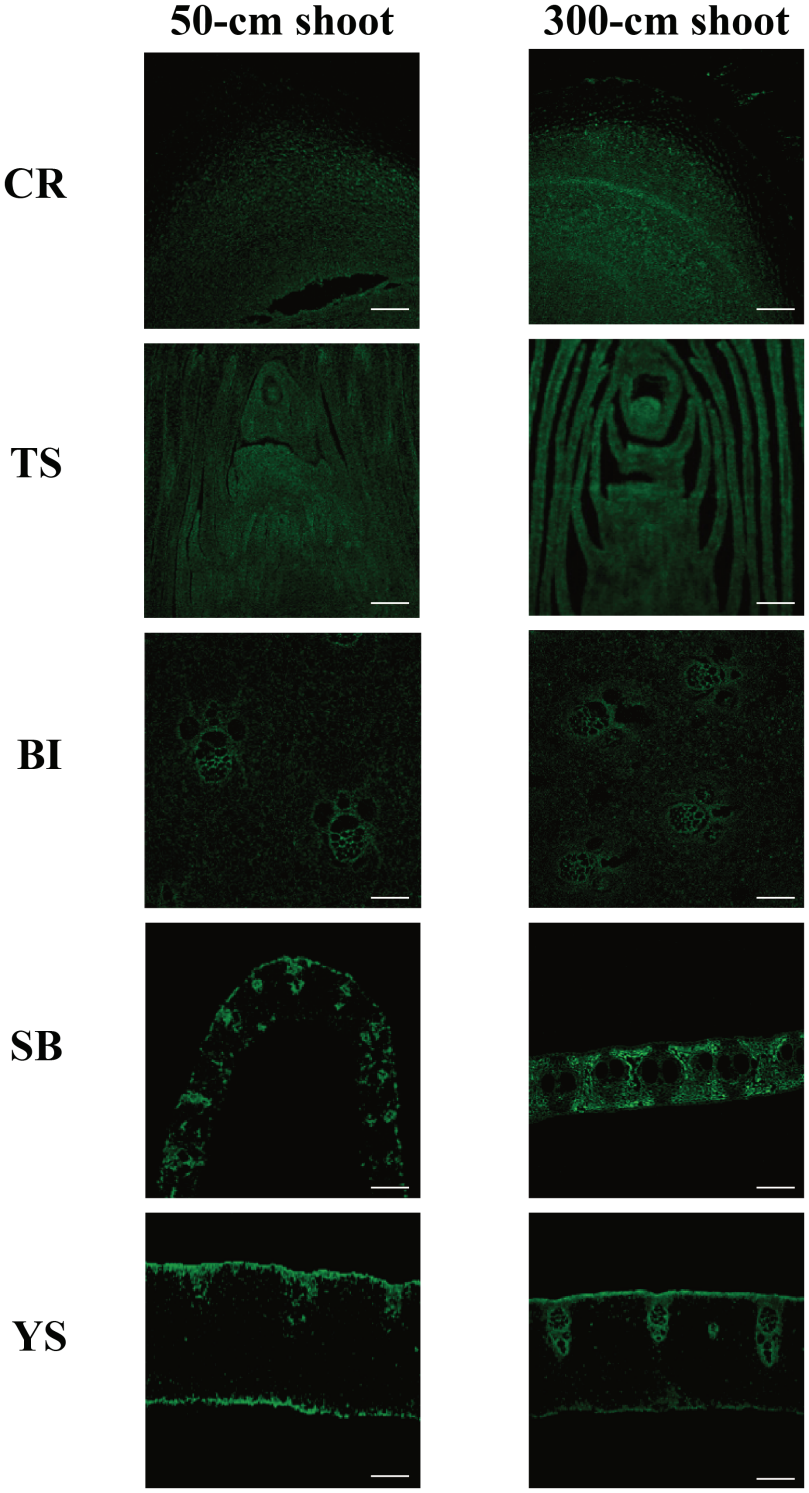
IAA immunofluorescence localization. CR root tip (the tip of the bamboo root growing out of the base of the bamboo shoot), TS tip of shoot, SB sheath blade (the upper component of culm sheaths, the base is connected to the tip of sheath and is the metamorphic leaf of bamboo shoot), BI bamboo shoot internode 1/5 from the tip, and YS young sheath. Bar = 50 μm.

Auxin was localized by immunostaining using IAA antibodies. Auxin was distributed in all young parts of the shoot. At the 50-cm shoot stage, auxin was mainly distributed in the division zone at the root tips, while auxin distribution in the root crown was significantly lower than that in the division zone (Figure 2). Auxin was mainly concentrated in the shoot tips and in the incompletely developed sheaths. In the culm sheath blade, culm sheaths, and internodes, auxin distribution was similar and mainly concentrated in the vascular region, while the thin-walled cells were less distributed with auxin observed between the vascular bundles. This may be related to the rapid cell division during the growth process. For the 300-cm-height bamboo shoots, with growth and development, the cell structure was more complete, and the distribution of auxin was similar to that of 50-cm bamboo shoots. Inside the culm sheath blade, the distribution of auxin was mainly in the thin-walled cells, while it was less in the vascular bundle cells. This may be because the growth and development of the culm sheaths, which act as metamorphic leaves, are completed, and the sheath blade is attached to them. The sheath blades gradually take on the function of the leaf as they develop. The distribution of starch also tends to stabilize, and after stabilization, its distribution area is consistent with that of auxin, exhibiting a mutual relationship (Supplementary Figure S2; Deng et al., 2020).

### Endogenous IAA Plays a Regulatory Role in Multiple Organs at Different Concentrations and Different Forms

**Figure 3:**
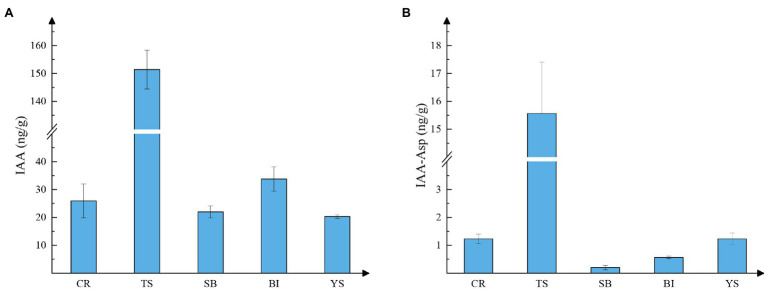
Auxin content of different tissues. CR root tip (the tip of the bamboo root growing out of the base of the bamboo shoot), TS tip of shoot, SB sheath blade (the upper component of culm sheaths, the base is connected to the tip of sheath and is the metamorphic leaf of bamboo shoot), BI bamboo shoot internode 1/5 from the tip, and YS young sheath. **(A)** Content of IAA; **(B)** Content of IAA-ASP (IAA-aspartate).

Because auxin distribution is similar in the pre-growth phase of shoots, we measured auxin content in the above-mentioned tissues of 300-cm shoots to determine the content of auxin in each tissue. High levels of free IAA were found in all tissues by combined high-performance liquid chromatography-mass spectrometry (HPLC-MS). The lowest levels in young culm sheaths reached 20.3 ng.g^−1^, and the highest levels in shoot tips reached 151.37 ng.g^−1^. The latter value was more than five times higher than the levels found in other tissues (Figure 3A). IAA conjugates of aspartate (IAA-Asp) were also detected in individual tissues (Figure 3B). The lowest levels were found in sheath blade and bamboo internodes, which may be because IAA is mainly stored as a conjugate and transported as active free IAA. Active IAA may be mainly transported. This indicates that auxin may play a regulatory role through local biosynthesis in the early stage of rapid growth of moso bamboo, mainly originating from the shoot tip.

### Spatio-Temporal Expression Pattern of *YUCCA* Gene Family Specific to Moso Bamboo

**Figure 4:**
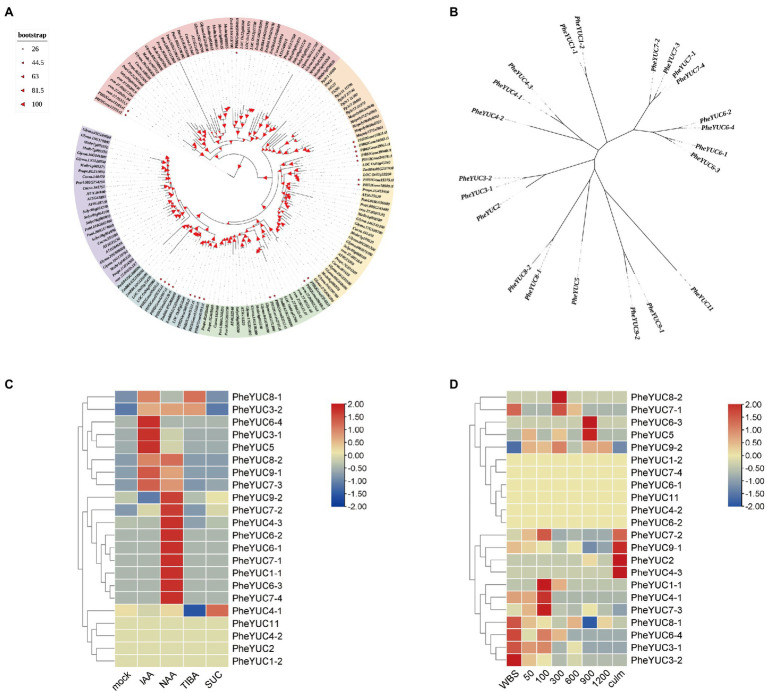
Phylogenetic tree and expression pattern analysis of *YUCCA* genes family. **(A)** Phylogenetic tree of 160 *YUCCA* genes of 14 species. Phe *Phyllostachys edulis*, AT *Arabidopsis thaliana*, Os *Oryza sativa*, evm *Amborella trichopoda*, Cucsa *Cucumis sativus*, Glyma *Glycine max*, Mapoly *Marchantia polymorpha*, Medtr *Medicago truncatula*, Pp *Physcomitrella patens*, Prupe *Prunus persica*, Potri *Populus trichocarpa*, Number *Selaginella moellendorffii*, Solyc *Solanum lycopersicum*, and Zm *Zea mays*. **(B)** Individual phylogenetic tree of 22 members of the moso bamboo *YUCCA* gene family. All genes were renamed with rice homology. Correspondence is shown in Supplementary Table S3. All trees are unrooted trees, and branch lengths represent only sequence differences. **(C,D)** Expression of different treatments and different growth stages (IAA Indole-3-acetic acid, NAA Naphthaleneacetic acid, TIBA Triiodobenzoic acid, and SUC Sucrose. WBS Winter bamboo shoots, 50 50-cm bamboo shoots, 100 100-cm bamboo shoots, 300 300-cm bamboo shoots, 600 600-cm bamboo shoots, 900 900-cm bamboo shoots, and 1,200 1,200-cm bamboo shoots). Spring bamboo shoots with good natural growth conditions and shoots 50 ± 2 cm tall were selected and injected with 20 μM IAA, 20 μM NAA, 200 μM TIBA, 200 μM SUC, and water 50 ml at the 5th internode and treated once every 3 days for a total of five times. Each treatment had 30 biological replicates. At the control shoot height of 4 m, the middle of the eighteenth node of each treatment was selected for analysis. The transcriptome data are available in the SAR database under the access number: PRJNA788576. The color scheme as in Log2 fold change relative to mock treatment.

*YUCCA* genes, which code for a key rate-limiting enzyme for auxin synthesis, have been used to measure auxin synthesis (Wang et al., 2019). Therefore, we identified 22 members of the moso bamboo YUCCA family using the Arabidopsis and rice YUCCA family sequence as a reference. It was identified as belonging to the FMO superfamily by the SMART and Pfam databases, and the annotated results are shown in Supplementary Table S3. We selected a total of 160 *YUCCA* genes from 14 species including moso bamboo to construct a phylogenetic tree. Moso bamboo is mainly related to monocots such as rice and maize, and we named moso bamboo-related genes according to their rice homology. We also constructed a phylogenetic tree of moso bamboo *YUCCA* genes and found that *YUCCA6*, which is related to leaf growth, and *YUCCA7*, which is related to drought stress, have multiple homologs in moso bamboo. There may be more important regulatory roles or redundancy of gene functions (Figure 4A; Kim et al., 2011; Lee et al., 2012). Naming the separate *YUCCA* genes of moso bamboo by relying on homology with rice and constructing a phylogenetic tree revealed that moso bamboo genes are more clustered and their functions may be more specific compared to rice (Figure 4B). Based on published data and transcriptome laboratory data, an analysis of family gene expression heat map by TBtools software revealed that some *YUCCA* genes have obvious processes in response to auxin and sugars, and their expression patterns are induced by them. For example, *PheYUC6-4*, *PheYUC3-1*, and *PheYUC5* clearly responded to IAA; *PheYUC9-2*, *PheYUC6-1*, and *PheYUC6-3* were induced by NAA, while *PheYUC7-3*, *PheYUC9-1*, and *PheYUC8-2* responded to both types of auxin. *PheYUC4-1* and *PheYUC8-1*were also clearly regulated by sucrose (Wang et al., 2017). For the different periods of moso bamboo shoot development, transcriptome analysis showed that the spatio-temporal expression pattern of *YUCCA* genes was also obvious (Figure 4C; Li et al., 2018a). The expression of most genes occurred in the pre-growth period, including *PheYUC3-1*, *PheYUC3-4*, and *PheYUC6-4*, while only *PheYUC5* and *PheYUC6-3* were expressed in the late period. *PheYUC9-2* is expressed throughout this stage, which may be due to the fact that auxin is mainly synthesized in large amounts in the pre-growth phase (Figure 4D).

**Figure 5:**
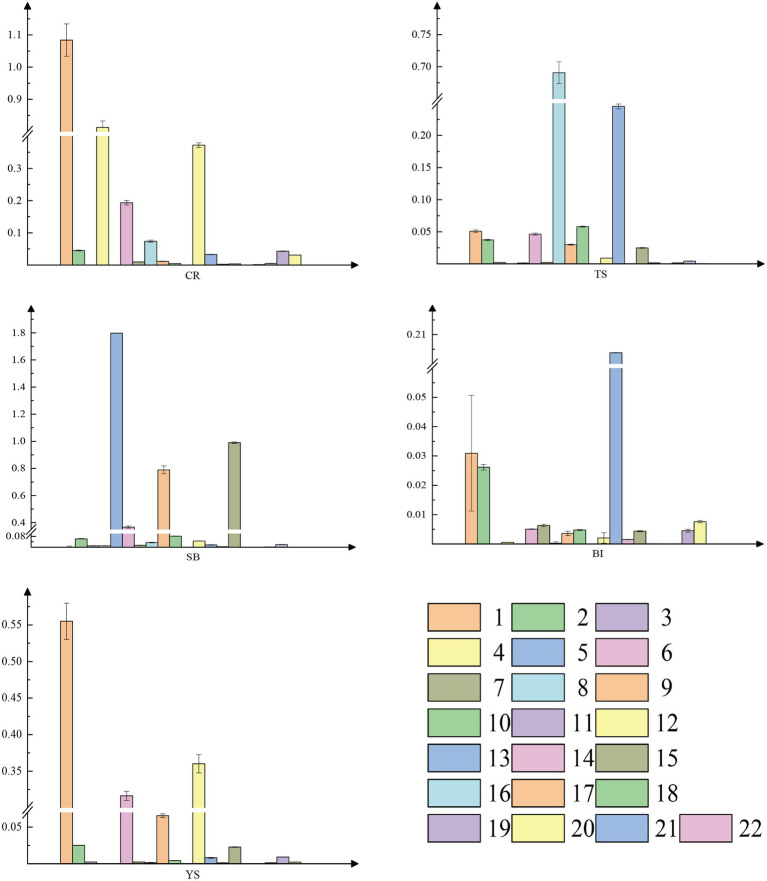
Real-time quantitative PCR(qRT-PCR) analysis of *YUCCA* gene family. 1 *PheYUC6-3*, 2 PheYUC9-1, 3 PheYUC2, 4 PheYUC4-2, 5 PheYUC6-1, 6 PheYUC5, 7 PheYUC4-1, 8 PheYUC3-2, 9 PheYUC1-2, 10 PheYUC8-1, 11 PheYUC6-2, 12 PheYUC9-2, 13 PheYUC3-1, 14 PheYUC4-3, 15 PheYUC1-1, 16 PheYUC8-2, 17 PheYUC7-4, 18 PheYUC7-2, 19 PheYUC7-1, 20 PheYUC7-3, 21 PheYUC11, and 22 PheYUC6-4. The reference gene is TIP41 of moso bamboo, see Supplementary Table S4 for relevant primer information. CR root tip (the tip of the bamboo root growing out of the base of the bamboo shoot), TS tip of shoot, SB sheath blade (the upper component of culm sheaths, the base is connected to the tip of sheath and is the metamorphic leaf of bamboo shoot), BI bamboo shoot internode 1/5 from the tip, and YS young sheath.

In contrast, tissue-specific expression of the genes was evident for different organs in the pre-rapid growth period (Figure 5). For example, *PheYUC6-3* was specifically expressed in roots, culm sheaths, and internodes, while *PheYUC9-2* was only expressed in the shoot tips. We performed a correlation analysis between *YUCCA* genes expression patterns and IAA contents in different tissues (Supplementary Figure S6). By calculating the Pearson correlation coefficients of IAA contents and *YUCCA* genes expression patterns in different tissues, we found that the genes we selected were consistent with the qRT-PCR results, and these genes showed significant positive correlation in specific tissues. These results suggest that there is spatial and temporal specificity and tissue specificity in the expression of *YUCCA* genes.

### Tissue-Specific Expression of *YUCCA* Genes

**Figure 6:**
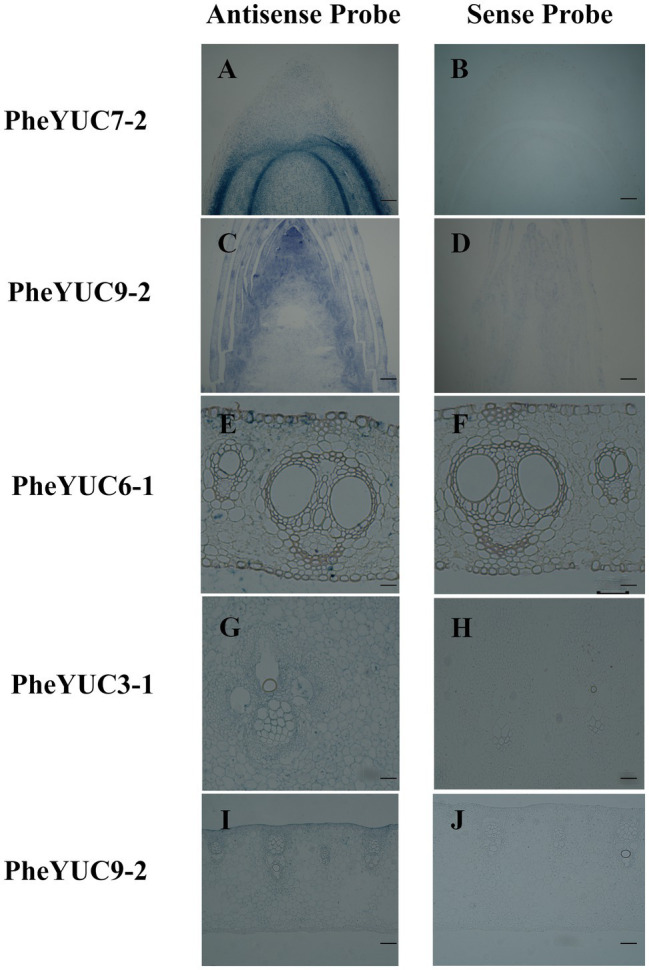
*In situ* expression analysis of *YUCCA* genes in different tissues. **(A,B)** root, **(C,D)** shoot tip, **(E,F)** sheath blade, **(G,H)** bamboo shoot internode 1/5 from the tip, and **(I,J)** young sheath. Bar = 50 μm.

Combining the evolutionary and expression pattern analyses, *PheYUC6-1, PheYUC9-1, PheYUC9-2, PheYUC3-1, PheYUC7-3*, and *PheYUC6-3* were selected for *in situ* hybridization analysis. There were significant differences in the spatio-temporal expression patterns of each gene (Figure 6; Supplementary Figure S3). *YUCCA* was highly expressed in the shoot tips, roots, and young sheath, while only one gene was weakly expressed in the sheath blade and bamboo internode. For example, *PheYUC6-1, PheYUC6-3, PheYUC9-2, PheYUC3-1*, and *PheYUC7-2* were expressed in the shoot tip, while only *PheYUC6-1* was expressed in the sheath blade. *PheYUC3-1* was expressed in the bamboo internodes, while *PheYUC6-3* was only expressed in the shoot tips and roots. These results verified that the shoot tip is the main site of auxin synthesis, and the auxin accumulation in the sheath blade and bamboo internode originates mainly from transport. Local biosynthesis of auxin exists in different tissues, and its accumulation helps to regulate the rapid growth process of moso bamboo.

## Discussion

### Rapid Growth of Moso Bamboo Is Regulated by Local Auxin Biosynthesis in Multiple Organs

Woody bamboo plants have a rapid growth pattern and similar to graminiferous annual monocotyledons such as maize, all determined by the common growth of multiple internodes, and each single internode may also have a consistent growth regulation mode (Wei et al., 2019; Gao et al., 2021; Wang et al., 2021). Therefore, we selected the most widely distributed and studied woody bamboo species, moso bamboo, to investigate the local auxin biosynthesis pattern and the effect on fast growth of woody bamboo. All young tissues of moso bamboo in the spring growth stage, including the roots, shoot tips, young culm sheaths, sheath blade, and internodes, were studied, and analysis showed that moso bamboo auxin is locally synthesized and that growth is regulated through multiple organs. Except for the sheath blade, the pattern of local auxin biosynthesis at different stages of pre-rapid growth is similar, occurring in actively dividing tissue cells, such as developing vascular tissues. Culm sheaths are a special metamorphic branching structure in bamboo, and the culm sheaths attached to culm are, by default, metamorphic leaf structures (Wang, 2017; Chen et al., 2021). As the sheath blade enters the rapid growth phase, they develop into more mature leaf structures with fully developed vascular bundles; so, most of the auxin is distributed on the walls of the thin-walled cells between the vascular bundles. This distribution highly overlaps the distribution of sugars.

### Sugar Signaling May Be Involved in Local Auxin Biosynthesis of Moso Bamboo

Sugar can promote auxin biosynthesis, and auxin and sugar signaling pathways can synergistically regulate growth (Sairanen et al., 2012; Li et al., 2020). In bamboo, sucrose, glucose, and starch distribution trends are similar, and these are distributed in the thin-walled cells surrounding mature vascular bundle cells (Wang et al., 2020). These results are consistent with the auxin distribution observed in this study. When the vascular bundle was not fully developed, auxin was distributed with the vascular bundle as the core, and when the vascular bundle was fully developed, auxin distribution was adjusted to be dominated by the thin-walled cells surrounding the vascular bundle (Supplementary Figure S3). This is consistent with the interaction of auxin and sugar signals to regulate the growth of stems in higher plants such as *Rosa hybrida* (Barbier et al., 2015; Mishra et al., 2021). The related *YUCCA* genes, such as *PheYUC9-2,* were also upregulated by glucose treatment, which further confirmed that the biosynthesis of auxin was closely related to a glucose signal (Figure 4B). These results reveal that the local biosynthesis of auxin may be initiated due to sucrose. When auxin is biosynthesized, it is transported to surrounding tissues to co-regulate growth with glycoconjugates. The presence of local biosynthesis in the higher auxin content in the internodal division zone may also be due to auxin transport after synthesis by other tissues such as the nearby sheath blade.

### Different *YUCCA* Genes Have Various Effects on Local Auxin Biosynthesis in Specific Organs

The genome-wide *YUCCA* gene family of moso bamboo was identified, and 22 *YUCCA* genes were obtained. Analysis of their expression patterns revealed that some genes were not involved in auxin synthesis, while others had obvious tissue-specific and spatio-temporal expression patterns. Phylogenetic tree analysis revealed that drought stress-related *YUCCA7* and leaf auxin synthesis-related *YUCCA6* in Arabidopsis have multiple homologs in moso bamboo, and they may have functional redundancy when combined with quantitative results (Figure 4; Kim et al., 2011; Lee et al., 2012; Wei et al., 2019). *In situ* hybridization revealed that different genes were expressed with obvious tissue specificity, mainly around actively dividing cells, consistent with auxin distribution. The genes that have a major synthetic function in the pre-growth phase of moso bamboo are *PheYUC3-1*, *PheYUC6-1*, *PheYUC6-3*, *PheYUC9-1*, *PheYUC9-2*, and *PheYUC7-3*. *PheYUC6-3* was expressed only in apical meristematic tissues such as shoot tips and roots, which may play a central role in auxin biosynthesis.

### Young Culm Sheaths Synthesize Auxin Involved in Rapid Growth Phytohormone Regulation of Moso Bamboo

Each node of bamboo attaches a culm sheath, grows, and senesces rapidly as the shoot grows. This unique interaction is important for growth and development. In addition to the high sugar content of culm sheaths, which regulate shoot growth with rapid senescence, the sheaths also serve as a control center for water and assimilate transport during the rapid growth of bamboo shoots. To investigate the effect of culm sheaths on the fast growth of moso bamboo, we also conducted stripping experiment and found that their growth slowed and tended to stop after partial stripping (Supplementary Figures S2, S5; Wang et al., 2020; Chen et al., 2021). However, the regulatory role of culm sheaths *via* phytohormones has not been explored. We found that culm sheaths have a high auxin content similar to their attached sheath blade. A large number of auxin synthesis genes (*YUCCA*) are expressed *in situ*, which appears to be inconsistent with their growth and senescence, while the auxin content of their attached culm divisions is not significant, and the related synthesis genes are weakly expressed. This suggests that the auxin required for their growth may come from the transport of auxin synthesized by the corresponding culm sheaths. This study is the first to detect auxin synthesis in culm sheaths. This knowledge fills a gap in understanding the regulation of auxin in culm sheaths, but experimental work is needed to determine the molecular mechanism and more work on transport molecules is needed to confirm the conclusion.

### The Auxin Required for Internode Elongation May Be Partly Derived From the Transport of Auxin Synthesized Elsewhere

We analyzed the local biosynthesis of auxin by measuring the IAA content and through *in situ* hybridization of key genes for auxin synthesis. Since the distribution of auxin was highly consistent at different developmental stages in the early stages, we selected different tissues of 300-cm shoots for our experiments and found that the shoot tip may be the main site of auxin synthesis, regulating growth through transport, while all other tissues had local biosynthesis, indicating that the spatial and temporal distribution of auxin was not caused by post-synthesis transport in a single shoot tip but was the result of local biosynthesis. However, for the sheath blade and bamboo internode, only the expression of a key biosynthetic gene was detected by *in situ* hybridization. The free IAA content was low too. The rapid growth of moso bamboo may be the result of multi-intersegmental co-growth, with cell division and differentiation in its meristematic zone determining intersegmental development. Transcriptome analysis revealed that auxin-related genes can play an important regulatory role (Wei et al., 2019). Although the interstitial meristem of each node is derived from the apical meristem, it is not functionally identical in terms of auxin synthesis. We suspect that it may be regulated by local biosynthetic auxin transport due to its attached young culm sheaths and shoot tips.

## Data Availability Statement

The original contributions presented in the study are included in the article/Supplementary Material, further inquiries can be directed to the corresponding author.

## Author Contributions

YB and JG designed the research. YB, MC, CM, WC, HZ, and ZC performed all experiments and analyzed the data. YB wrote the original manuscript. YB, SM, JL, and JG proofread the manuscript. All authors contributed to the article and approved the submitted version.

## Funding

This study was supported by Fundamental Research Funds of ICBR (1632020005 and 1632021017).

## Conflict of Interest

The authors declare that the research was conducted in the absence of any commercial or financial relationships that could be construed as a potential conflict of interest.

## Publisher’s Note

All claims expressed in this article are solely those of the authors and do not necessarily represent those of their affiliated organizations, or those of the publisher, the editors and the reviewers. Any product that may be evaluated in this article, or claim that may be made by its manufacturer, is not guaranteed or endorsed by the publisher.

## Footnotes

^1^ http://www.arabidopsis.org/

^2^ http://rice.plantbiology.msu.edu/index.shtml

^3^ http://forestry.fafu.edu.cn/db/PhePacBio/index.php

^4^ http://pfam.xfam.org

^5^ https://www.ncbi.nlm.nih.gov/Structure/cdd/wrpsb.cgi

^6^ http://www.megasoftware.net/mega.php
