# Supplementary material for: New Insights Into the Local Auxin Biosynthesis and Its Effects on the Rapid Growth of Moso Bamboo (Phyllostachys edulis)
Source: Front Plant Sci. 2022 May 3;13:858686. doi: 10.3389/fpls.2022.858686 (PMC9111533; doi:10.3389/fpls.2022.858686)
Supplement: Supplementary file 1 [file Data_Sheet_1.ZIP › Datasheet 1_v1/Supplementary Charts/Supplementary Charts/Figure S4.XIC diagram of different tissues of metabolites.docx]

**
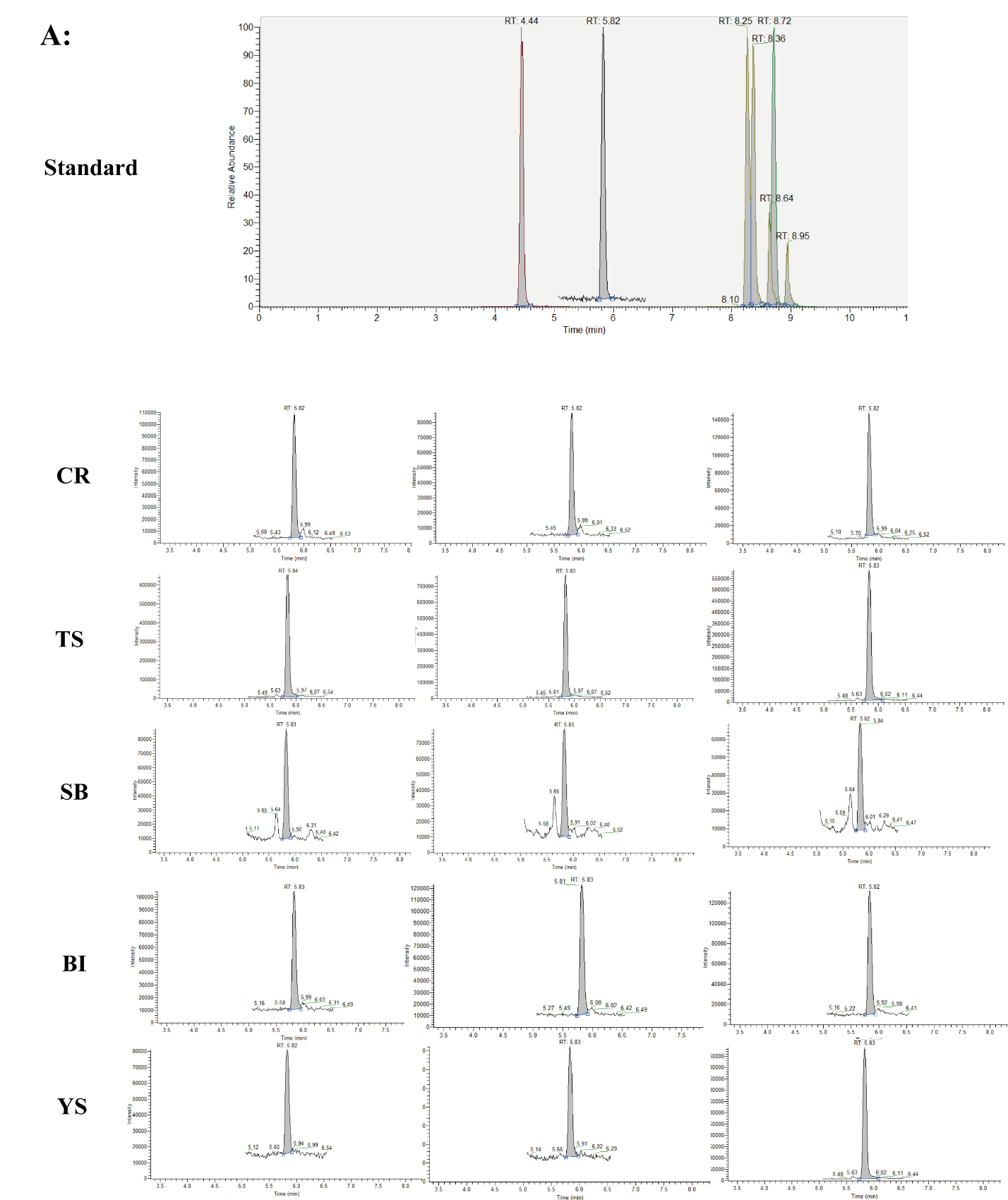
**

**
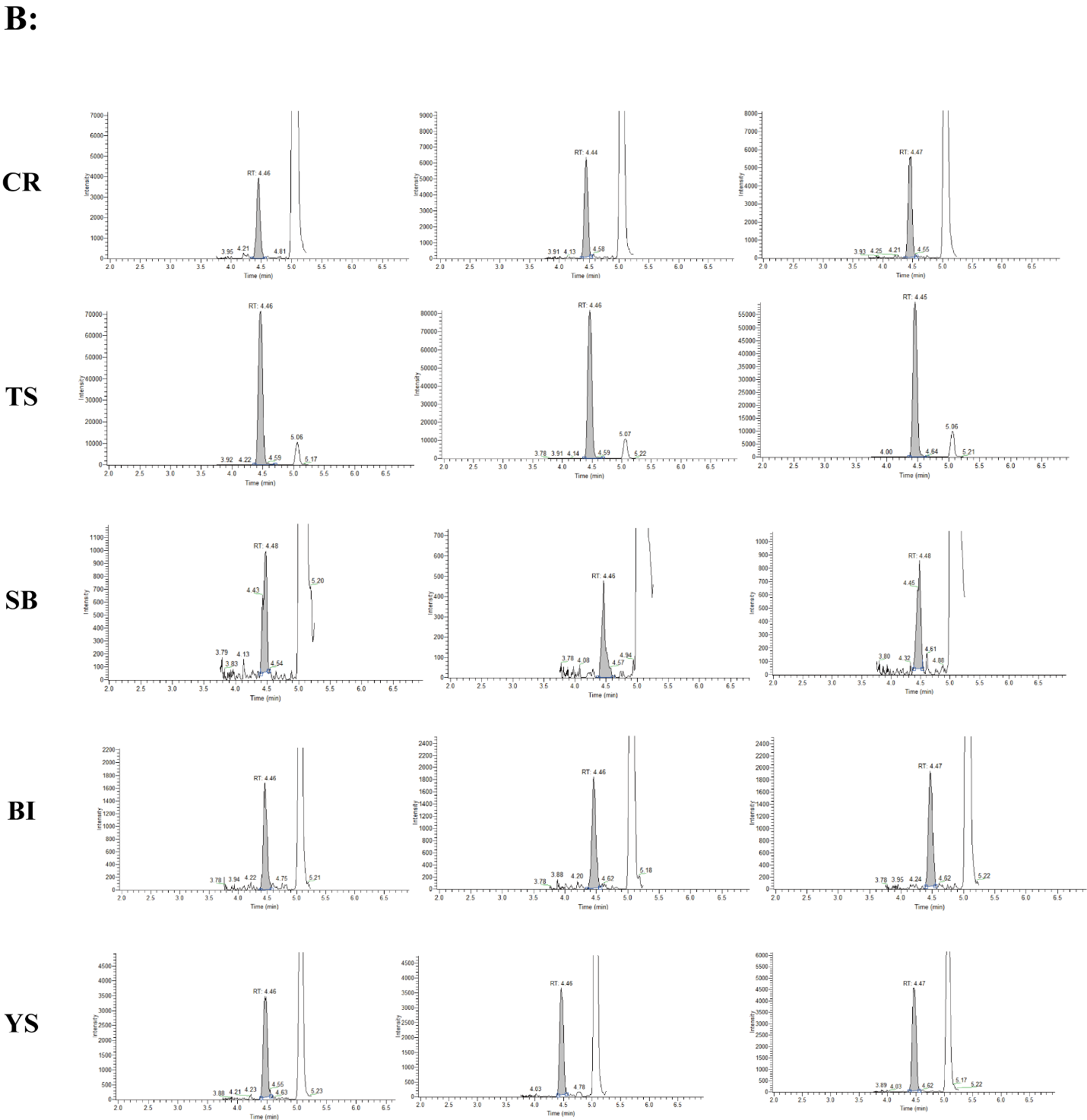
**

**Figure S3.** XIC diagram of different tissues of metabolites. (A) IAA, (B) IAA-aspartate, ( IAA-ASP ) CR root, TS tip of shoot, SB sheath blade, BI bamboo shoot internode 1/5 from the tip, YS young sheath.
