# Supplementary material for: New Insights Into the Local Auxin Biosynthesis and Its Effects on the Rapid Growth of Moso Bamboo (Phyllostachys edulis)
Source: Front Plant Sci. 2022 May 3;13:858686. doi: 10.3389/fpls.2022.858686 (PMC9111533; doi:10.3389/fpls.2022.858686)
Supplement: Supplementary file 1 [file Data_Sheet_1.ZIP › Datasheet 1_v1/Supplementary Charts/Supplementary Charts/Figure S6 Analysis of the correlation between YUCCA genes and IAA contents.docx]

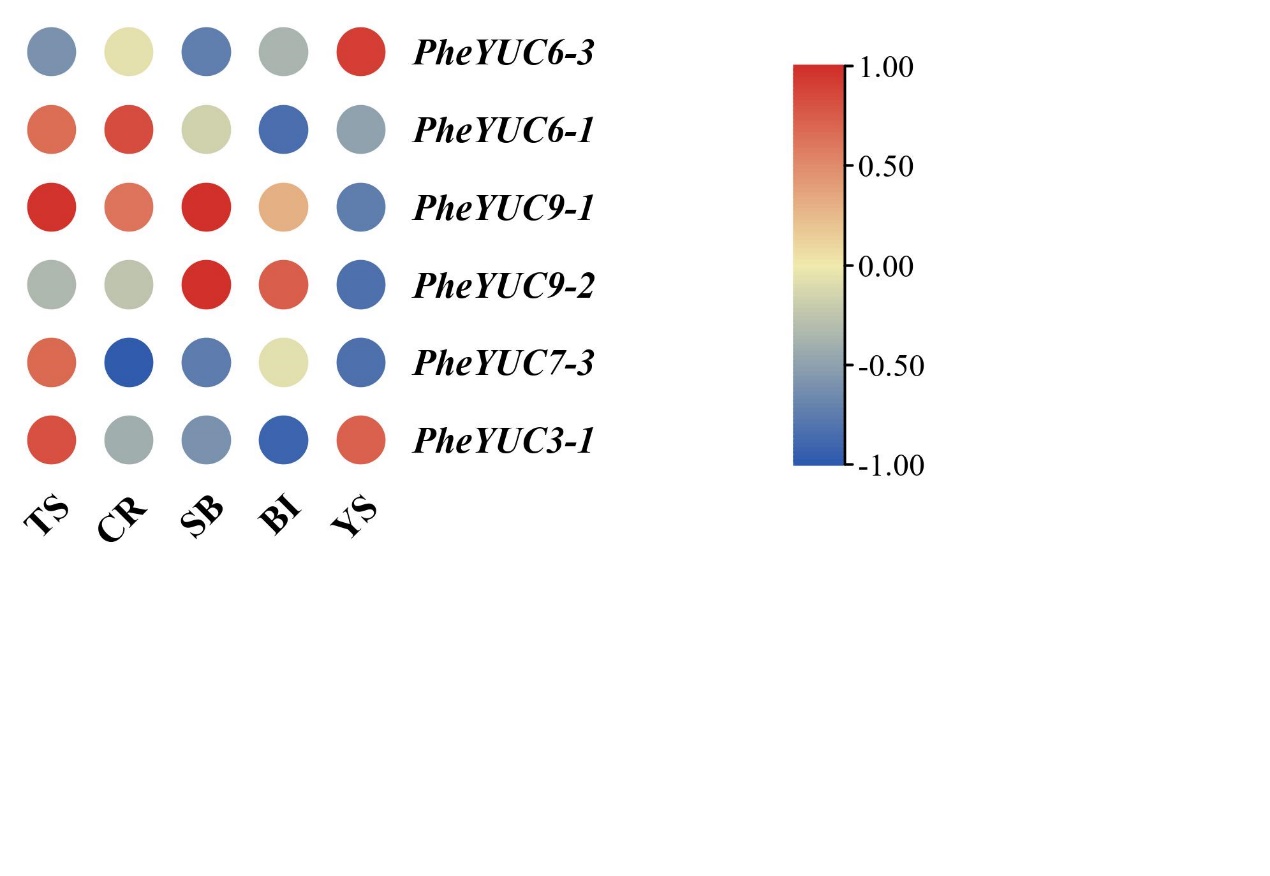


**Figure S6** Analysis of the correlation between *YUCCA* genes and IAA contents. **Notes** The Pearson correlation coefficient between IAA contenst of each sample and *YUCCA* genes were calculated and visualized by TBtools software.
