# Supplementary figures and images for: New Insights Into the Local Auxin Biosynthesis and Its Effects on the Rapid Growth of Moso Bamboo (Phyllostachys edulis)
Source: Front Plant Sci. 2022 May 3;13:858686. doi: 10.3389/fpls.2022.858686 (PMC9111533; doi:10.3389/fpls.2022.858686)

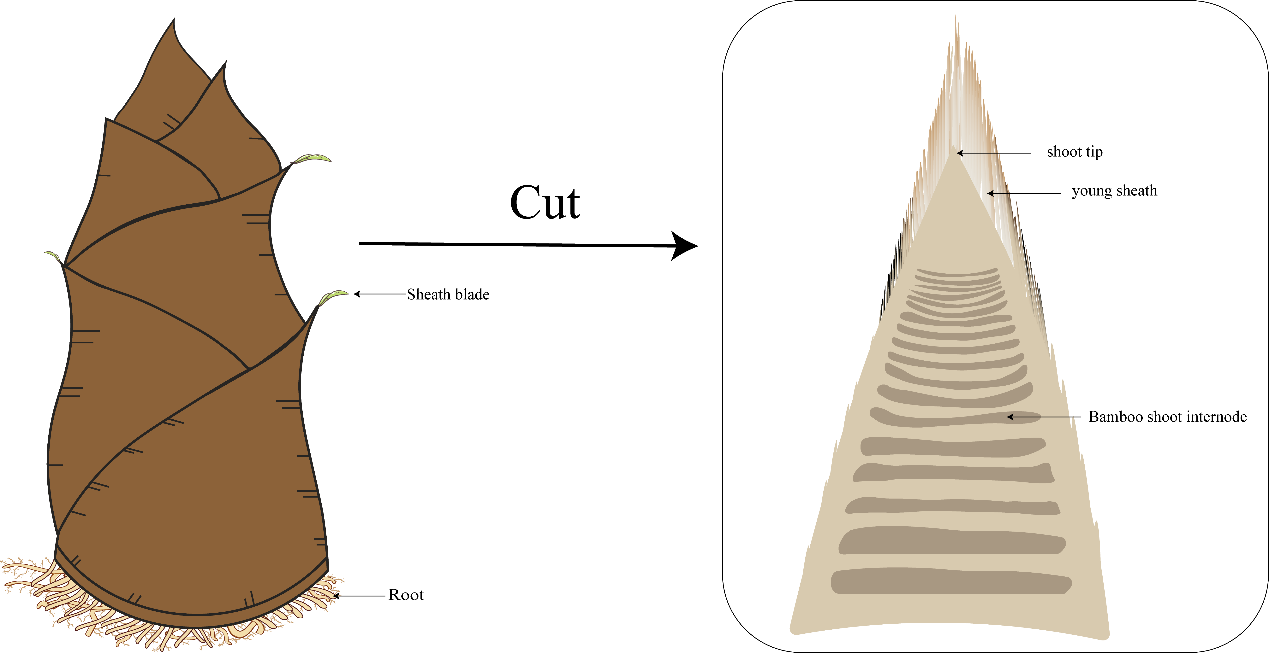


**Figure S1.** Schematic diagram of different sampling sites

Supplement: Supplementary file 1 [file Data_Sheet_1.ZIP › Datasheet 1_v1/Supplementary Charts/Supplementary Charts/Figure S1 Schematic diagram of different sampling sites.docx]

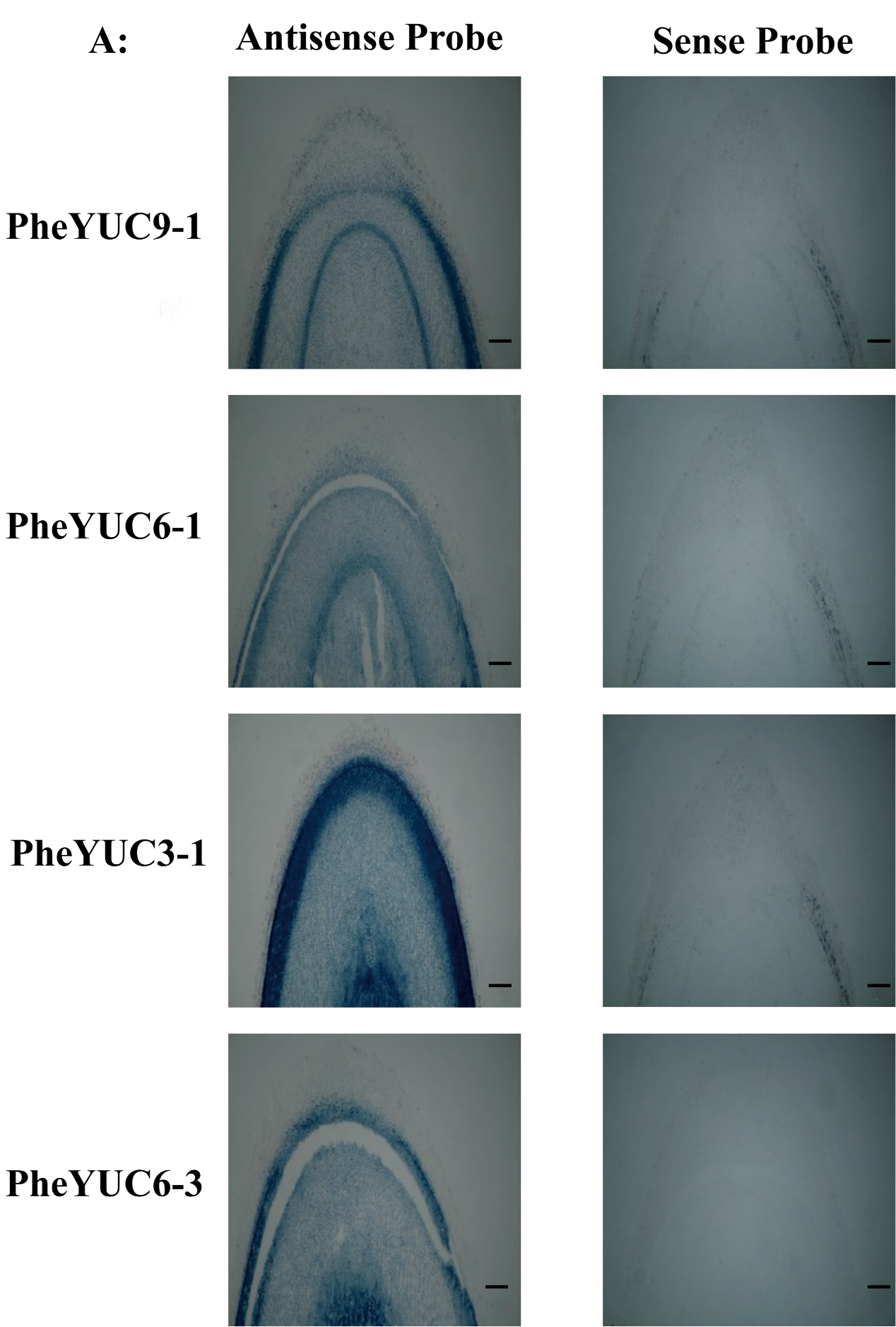


**
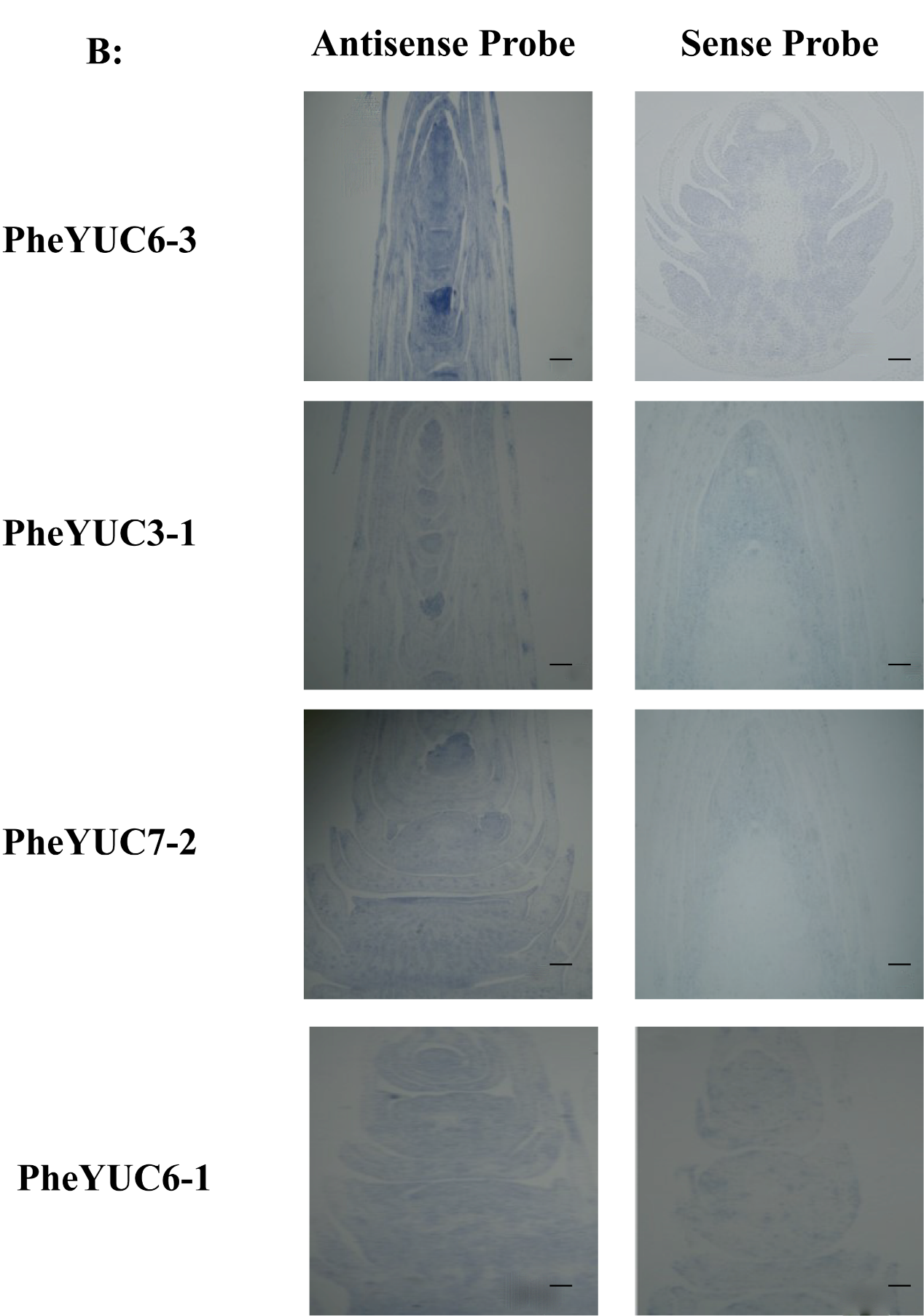
**


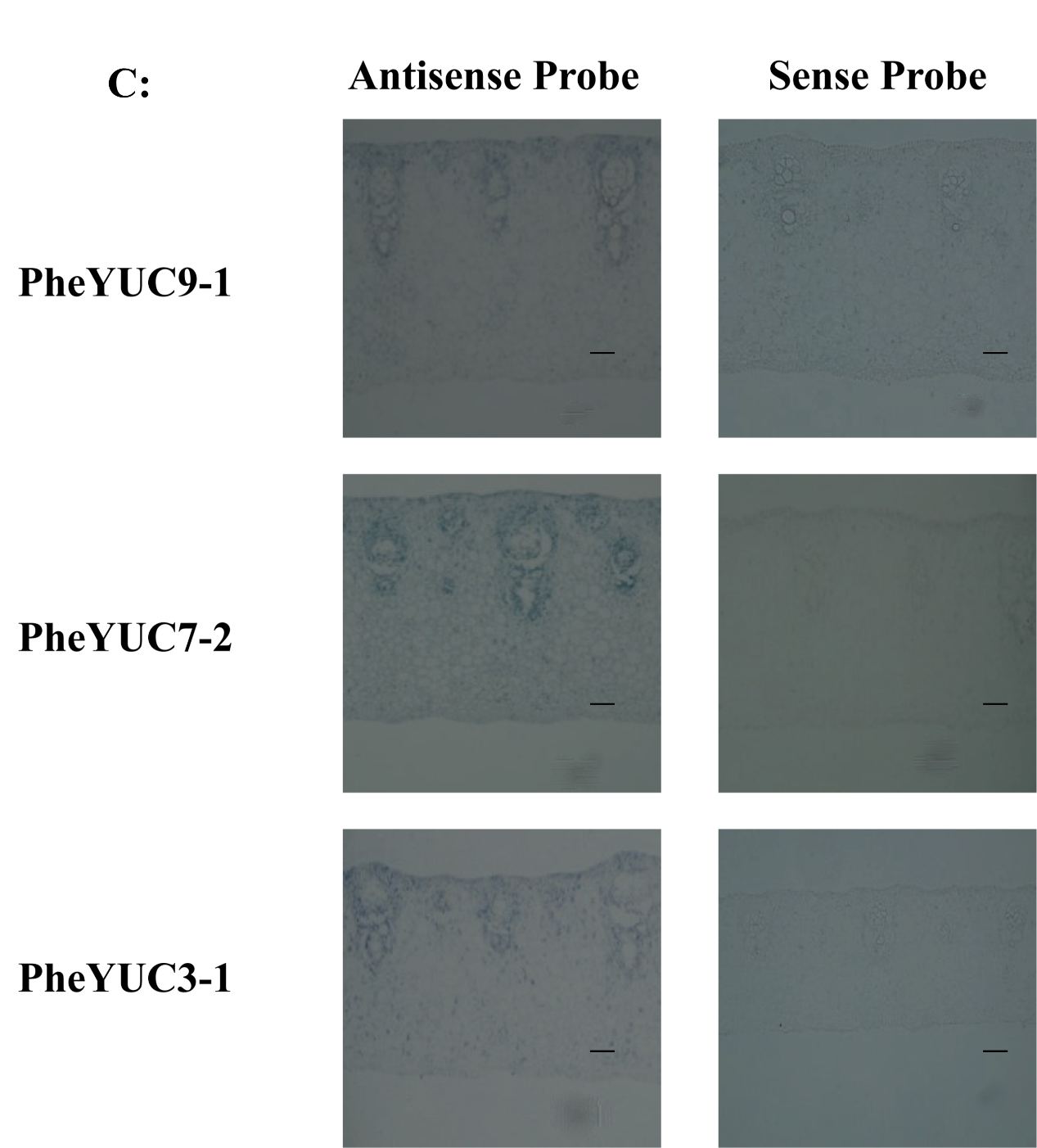


**Figure S3.** In situ expression of other key YUCCA genes. (A) root, (B) shoot tip, (C) young sheath. Bar = 50 μm

Supplement: Supplementary file 1 [file Data_Sheet_1.ZIP › Datasheet 1_v1/Supplementary Charts/Supplementary Charts/Figure S3 In situ expression of other key YUCCA genes.docx]

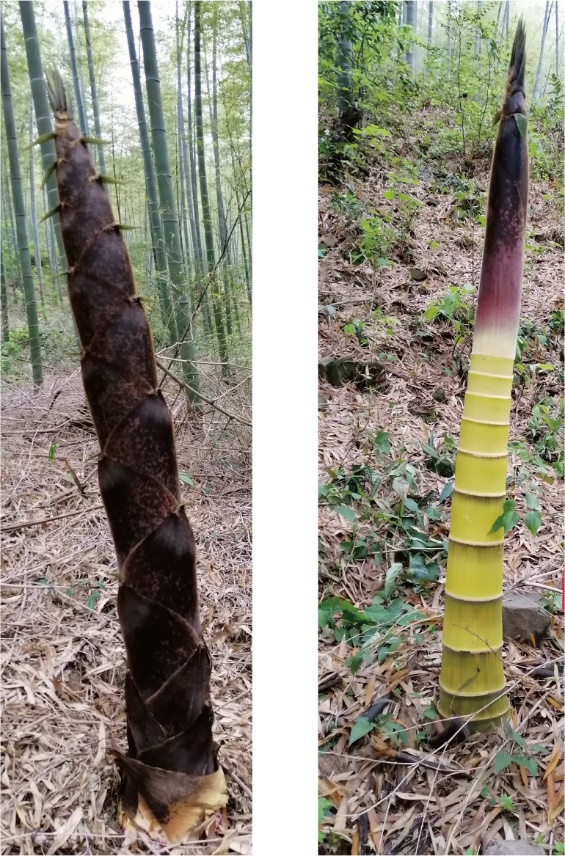


**Figure S5.** Experiment of sheath stripping of bamboo shoots

Supplement: Supplementary file 1 [file Data_Sheet_1.ZIP › Datasheet 1_v1/Supplementary Charts/Supplementary Charts/Figure S5 Experiment of sheath stripping of bamboo shoots.docx]
